# Moderate ethanol exposure during early ontogeny of the rat alters respiratory plasticity, ultrasonic distress vocalizations, increases brain catalase activity, and acetaldehyde-mediated ethanol intake

**DOI:** 10.3389/fnbeh.2022.1031115

**Published:** 2022-11-10

**Authors:** Genesis D’aloisio, María Belén Acevedo, Asier Angulo-Alcalde, Verónica Trujillo, Juan Carlos Molina

**Affiliations:** ^1^Laboratorio de Alcohol, Ontogenia y Aprendizaje, Instituto de Investigación Médica Mercedes y Martín Ferreyra (INIMEC-CONICET-UNC), Córdoba, Argentina; ^2^Facultad de Psicología, Universidad Nacional de Córdoba, Córdoba, Argentina; ^3^Departamento de Procesos Psicológicos Básicos y su Desarrollo, Facultad de Psicología, Universidad del País Vasco (UPV/EHU), Donostia-San Sebastián, Spain; ^4^Laboratory of Neuroendocrinology, Department of Biophysics, Escola Paulista de Medicina, Universidade Federal de São Paulo, São Paulo, Brazil; ^5^Departamento de Fisiología, Facultad de Ciencias Exactas, Físicas y Naturales, Universidad Nacional de Córdoba, Córdoba, Argentina

**Keywords:** ethanol, acetaldehyde, breathing, ultrasonic vocalization, ethanol intake, catalase, early ontogeny

## Abstract

Early ontogeny of the rat (late gestation and postnatal first week) is a sensitive period to ethanol’s positive reinforcing effects and its detrimental effects on respiratory plasticity. Recent studies show that acetaldehyde, the first ethanol metabolite, plays a key role in the modulation of ethanol motivational effects. Ethanol brain metabolization into acetaldehyde via the catalase system appears critical in modulating ethanol positive reinforcing consequences. Catalase system activity peak levels occur early in the ontogeny. Yet, the role of ethanol-derived acetaldehyde during the late gestational period on respiration response, ultrasonic vocalizations (USVs), and ethanol intake during the first week of the rat remains poorly explored. In the present study, pregnant rats were given a subcutaneous injection of an acetaldehyde-sequestering agent (D-penicillamine, 50 mg/kg) or saline (0.9% NaCl), 30 min prior to an intragastric administration of ethanol (2.0 g/kg) or water (vehicle) on gestational days 17–20. Respiration rates (breaths/min) and apneic episodes in a whole-body plethysmograph were registered on postnatal days (PDs) 2 and 4, while simultaneously pups received milk or ethanol infusions for 40-min in an artificial lactation test. Each intake test was followed by a 5-min long USVs emission record. On PD 8, immediately after pups completed a 15-min ethanol intake test, brain samples were collected and kept frozen for catalase activity determination. Results indicated that a moderate experience with ethanol during the late gestational period disrupted breathing plasticity, increased ethanol intake, as well brain catalase activity. Animals postnatally exposed to ethanol increased their ethanol intake and exerted differential affective reactions on USVs and apneic episodes depending on whether the experience with ethanol occur prenatal or postnatally. Under the present experimental conditions, we failed to observe, a clear role of acetaldehyde mediating ethanol’s effects on respiratory plasticity or affective states, nevertheless gestational acetaldehyde was of crucial importance in determining subsequent ethanol intake affinity. As a whole, results emphasize the importance of considering the participation of acetaldehyde in fetal programming processes derived from a brief moderate ethanol experience early in development, which in turn, argues against “safe or harmless” ethanol levels of exposure.

## Introduction

Evidence from clinical and preclinical studies indicates that chronic exposure to high doses of ethanol during the gestational period is capable of generating craniofacial dysmorphologies, neuropathological alterations, and cognitive deficits (Wozniak et al., 2019; Almeida et al., 2020). These features constitute a permanent clinical condition known as Fetal Alcohol Syndrome (FAS) (Lemoine, 1968; Jones et al., 1973; Burd et al., 2004; O’Leary, 2004; Wozniak et al., 2019), while a broader array of outcomes are known as Fetal Alcohol Spectrum Disorders (FASD) (Riley and Mcgee, 2005; Mattson et al., 2019). Furthermore, chronic intrauterine exposure to the drug in humans also represents a risk factor for Sudden Infant Death Syndrome (SIDS) (Iyasu et al., 2002; Burd et al., 2004; Bailey and Sokol, 2011; O’Leary et al., 2013) and for hypoxic ischemic effects upon the developing brain (Pillekamp et al., 2007).

Beyond ethanol’s teratogenic effects, early brief experiences (e.g., gestational days, GDs 17–20) with low-to-moderate doses of the drug (e.g., sub-teratogenic, 0.5–2.0 g/kg) recruit sensory, perceptual, and learning capabilities of the organisms that result in the recognition of its chemosensory attributes, sensitivity to its motivational properties, and the subsequent drug seeking and consumption (Chotro et al., 2007; Molina et al., 2007; Abate et al., 2008). The process has been recently proposed as an early programming of alcohol affinity (Miranda-Morales et al., 2020). The late gestation and the first week of the rat are stages analogous to the second and third gestational human trimester in terms of brain growth (Dobbing and Sands, 1979; Tran et al., 2000; Almeida et al., 2020). Throughout these periods, the fetus and neonate rats detect and discriminate the chemosensory compounds (odor and taste) of ethanol in the amniotic fluid and the milk of their dam. Furthermore, the physiological and reinforcing effects of the drug promote learning processes which in turn lead to an increased preference and affinity of the drug (Domínguez et al., 1996, 1998; Abate et al., 2000, 2002, 2008). Such early experiences not only generate sensitivity to the positive motivational effects of ethanol but also sensitize the organism to the consequences of ethanol upon respiratory plasticity. Brief experiences with moderate doses of ethanol during late gestation (Cullere et al., 2015) or within the first postnatal week that generate respiratory disruptions indicative of sensitization processes (Cullere et al., 2015; Macchione et al., 2016, 2018, 2021; Acevedo et al., 2017a; D’aloisio et al., 2020). Moreover, the association of a salient odor stimulus (e.g., ethanol) in the presence of ethanol-related respiratory disruptions, elicit later conditioned breathing depressions and increase the occurrence of apneic episodes (Cullere et al., 2015; Macchione et al., 2016, 2018).

Considerable research indicates that ethanol’s positive and negative motivational effects are key factors in the determination of seeking behavior, consumption, and later misuse of the drug (Cunningham et al., 2000). In terms of ethanol intake, the mere exposure to the drug prenatally, exacerbates pup’s consumption of milk mixed with ethanol without affecting the intake of milk as a sole liquid at PD 3 (Pueta et al., 2008), increases the intake of either ethanol, and ethanol taste equivalent such as sucrose-quinine at PD 14 (Domínguez et al., 1998), and reduces the aversive consequences of high ethanol doses during adolescence (Fabio et al., 2013, 2015). It is interesting to note that, during the first or second week of life, pups show a great ethyl affinity in terms of voluntary or forced intake of ethanol, which reaches the maximum values compared to other periods of development and does not require initiation procedures that are necessary in adults to occur (Truxell and Spear, 2004; Sanders and Spear, 2007). Increased ethanol intake was also observed during PDs 7 and 11 as a function of the nursing context (Miranda-Morales et al., 2016) and as a result of repeated exposure to a high ethanol dose (3.0 g/kg) during neonatal period at PD 11 (Acevedo et al., 2017a) when using an artificial intake test. However, it is unknown whether an experience with ethanol during the late gestation would increase ethanol intake using the artificial intake test and induce respiratory alterations shortly after birth.

In addition to ethanol’s positive reinforcing effects, alcohol exposure sensitizes the organism to its anxiolytic/anxiogenic effects. Chronic exposure to high (36% *ad libitum*) and moderate (0.6 g/kg) ethanol doses throughout gestation alter the hypothalamic-pituitary-adrenal (HPA) axis tone, producing a long-term hyperresponsiveness to stressors in rats and monkeys (Schneider et al., 2002; Weinberg et al., 2008; Hellemans et al., 2010). The intragastric experience with low doses of ethanol (0.25, 0.5, or 1.25 g/kg) during PD 14 reduces an odor avoidance response at PD 15 (Pautassi et al., 2006) and increases the time spent in the illuminated area in a light/dark box test with a 0.5 g/kg ethanol dose at PD 16 (Miranda-Morales et al., 2014). Recent studies also suggested that early anxiolytic effects of ethanol, as apneic episodes during PDs 3–7 are reduced after repeated intracisternal administrations of a high ethanol dose (300 mg%, Trujillo et al., 2020) and after intragastric administrations of low or moderate ethanol doses (0.75, 1.37, and 2.0 g/kg) during PDs 3–9 (D’aloisio et al., 2020; Anunziata et al., 2021). Binge-like ethanol exposure (3.0 g/kg) during PDs 4–10 decreases ultrasonic vocalizations (USVs) – considered as a proxy of anxiety- on PD 14 (Barron and Gilbertson, 2005) and a low dose of ethanol (0.5 g/kg) exerts a protected effect on maternal separation stress-induced vocalizations on PD 14 (Pautassi et al., 2007). Exposure to a high ethanol dose (ethanol vapor levels: 7.8 ± 0.13 g/dl) during PDs 3–5 increases anxiety-like behaviors in an elevated plus maze during adolescence (Baculis et al., 2015). Vocalizations are believed to provide an index of the affective state of the animal (Knutson et al., 2002; Portfors, 2007; Simola, 2015). This information is important for understanding the behaviors or drug effects of animals whether in a natural or experimental environment. When examining the affective consequences of ethanol upon the expression of apneic episodes and maternal separation-induced distress vocalizations, little attention has been paid during the late gestational period. Indeed, in contrast to the well-defined appetitive and aversive USVs in adults (i.e., appetitive: “50-kHz vocalizations” range 30–100 kHz, Knutson et al., 2002; Simola and Brudzynski, 2018a,b, aversive: “20-kHz vocalizations” range 18–32 kHz, Borta et al., 2006), pups’ vocalizations are poorly studied and limited to two frequency ranges (40-kHz and 60-kHz) despite being capable of emitting a wide range of these (10—180 KHz, 2–200 KHz; Brudzynski et al., 1999; Kaidbey et al., 2019; Potasiewicz et al., 2020).

As described above, early experience with ethanol generates high affinity toward its positive effects that facilitates ethanol consumption later on. In fact, it seems to be a sensitive period in the perception of ethanol positive effects. When pups are exposed to a high ethanol dose (3.0 g/kg) during PDs 7–8, ethanol palatability and consumption increase 3 days later, but if this experience occurs during PDs 10–11 the opposite effects are observed, for instance, disgust reactions and decreased consumption (Arias and Chotro, 2006). A possible explanation of the discrepancy might be related to the changes in terms of ethanol metabolism that occur during this period of brain maturation (March et al., 2013a). In adults, peripherical accumulation of the first ethanol metabolization product (acetaldehyde; ACD) induces aversive consequences such as conditioned taste aversion, conditioned place aversion, reduction of locomotor activity, and self-administration of the drug (Aragon et al., 1986, 1991a; Myers et al., 1987; Kunin et al., 2000; Quintanilla et al., 2002; Escarabajal et al., 2003; Quintanilla and Tampier, 2003; Quertemont et al., 2004; McLaughlin et al., 2008) whereas acetaldehyde central accumulation is associate with ethanol’s reinforcing effects such as increment of consumption, anxiolytic effects, increase of locomotor activity, and self-administration of the drug (Amit et al., 1977; Brown et al., 1979; Smith et al., 1984; Arizzi et al., 2003; Correa et al., 2003, 2009; Arizzi-La France et al., 2006). Interestingly, catalase system peak levels of activity, which is mainly involved in the metabolization of ethanol into acetaldehyde in the brain, negatively correlates with age. In fact, the activity of this enzymatic system is almost eight times higher during early ontogeny than during adolescence and adulthood (Del Maestro and McDonald, 1987). On the contrary, given the hepatic immaturity in the periphery, the capacity of fetuses and neonates to metabolize ethanol into acetaldehyde is markedly lower compared to adolescent and adult rats (Kelly et al., 1987). This metabolic profile that involves peripheral and central production of ethanol’s first metabolite critically, modulates the sensitivity to the motivational effects of ethanol and the amount of ethanol self-administrated (Chao, 1995). In newborn rats, the central administration of low doses of ethanol (100 mg%) or acetaldehyde (0.35 μmol) generates appetitive conditioning assessed by the artificial nipple technique (Nizhnikov et al., 2006; March et al., 2013b,c). Moreover, these early ethanol’s appetitive memories are blocked when inhibiting the catalase system activity using sodium azide (Nizhnikov et al., 2007) or when sequestering brain acetaldehyde via D-penicillamine (March et al., 2013b) in newborn pups. The use of D-penicillamine also inhibited conditioned place preference induced by ethanol on PD 15 (Pautassi et al., 2011). Recently, it has been reported that the absence of prenatal acetaldehyde via D-penicillamine decreases ethanol intake during the second postnatal week (Gaztañaga et al., 2017). Nevertheless, to our knowledge, the role of acetaldehyde in the early modulation of ethanol’s affective effects has not been analyzed yet. In addition, little is known about the possible contribution of acetaldehyde in the mediation of early ethanol respiratory consequences.

The major aims of the present study were threefold: (i) to determine whether a brief experience with a moderate dose of ethanol during the late gestation and/or the rat’s first week of life affect their respiratory and affective state response and subsequent ethanol intake, (ii) to evaluate if the absence of acetaldehyde during the late gestation via D-penicillamine modulates ethanol’s effects in pups; such as the respiratory and affective response, and subsequent ethanol intake as well, (iii) to examine whether central catalase is increased in pups’ brains as experienced a moderate dose of ethanol during late gestation.

## Materials and methods

### Subjects

A total number of 260 Wistar-derived pup rats, representative of 35 litters, were employed for all the evaluations performed in the present study. Rats were born and reared at the vivarium of the Instituto de Investigación Médica Mercedes y Martín Ferreyra (INIMEC-CONICET-UNC, Argentina). The animal colony room was illuminated on a 12 h light/dark cycle (lights on: 08:00–20:00 h) at an ambient temperature 22–24°C and humidity of ∼45%. On the day of proestrus, females (body weights: 200–250 g) were housed overnight with males. Vaginal smears were checked the following morning. The day of sperm detection was designated gestational day 0 (GD 0) and the day of parturition postnatal day 0 (PD 0). At PD 1, each litter was randomly culled to 10 pups (five males and five females, whenever possible). Pups were kept with their dams in standard cages that contained tap water and normal chow *ad libitum* (ACA Nutrición, Buenos Aires, Argentina). All experimental treatments and animal’s maintenance were in compliance with the Guide for Care and Use of Laboratory Animals (National Research Council, 2011) and received the Institutional Animal Care and Use Committee (CICUAL-INIMEC-CONICET-UNC) approval. To minimize confounds between litter and treatment effects no more than one male and one female per litter were assigned to the same experimental condition (Holson and Pearce, 1992).

### Drug treatments and drug preparations during late gestation

Thirty-five dams were used and daily handled to reduce the stress associated to the experimental manipulation, and the potential source of unexplained variation among animals. From GDs 17–20 dams received subcutaneous (sc.) administrations near their neck of either D-penicillamine (50 mg/kg) or saline (0.9% NaCl in distilled water) 30 min before receiving intragastric (ig.) administrations of either 2.0 g/kg ethanol or water (tap water) every day. The dose employed here results in peak blood and brain ethanol concentrations equivalent to 220 and 180 mg/dL, respectively (Cullere et al., 2015; Macchione et al., 2016). Following the final treatment (GD 20) the dams remained undisturbed until parturition.

D-penicillamine was obtained from Sigma-Aldrich, Steinheim, Germany and dissolved at a concentration of 7.5 mg/ml in saline (0.9% v/v). The 50 mg/kg dose selected reduces the reinforcing effects of ethanol in infant and neonate rats (Pautassi et al., 2011; Gaztañaga et al., 2017). The injection volume was 0.07 μl/g and was administered within ∼4 s. Ethanol dose (2.0 g/kg) was achieved by administering 0.015 ml/g of a 16.8% (v/v) ethanol solution (Porta Hnos, Cordoba, Argentina). An equivalent volume of tap water was administered as vehicle (0.0 g/kg).

### Experimental procedures during postnatal days

**FIGURE 1:**
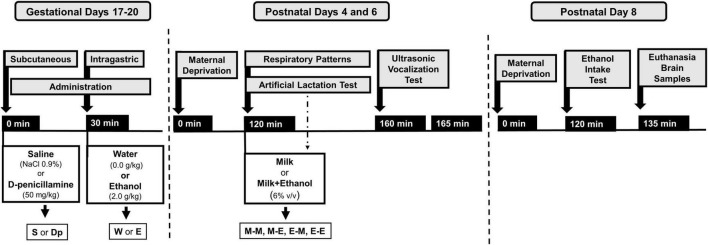
Experimental procedures performed during gestational days 17–20 and postnatal days 4 and 6. The information provided here describes drug treatments during gestation (administration: saline or D-penicillamine; water or ethanol), postnatal daily temporal course concerning maternal isolation (120 min), postnatal assessments: a 40-min long artificial lactation test and respiratory patterns recording simultaneously followed by 5-min ultrasonic vocalization test. During artificial lactation tests, animals received intraoral infusion of milk (M) or milk mixed with ethanol (E). Four groups were defined as a result of these interventions: M-M, M-E, E-M, and E-E. Overall, 16 groups were defined as a function of prior drug treatment during late gestation and postnatally. For more details, please see section of “Materials and methods”.

At PDs 4 and 6, pups were removed from their maternal cages and placed in pairs. Pups were then exposed to intraoral infusions of either milk or milk mixed with ethanol while their breathing frequencies and apneic episodes were assessed in a whole-body plethysmograph (Pleth) for 40 min. Final data was averaged across 15 bins of evaluation. Assessments were followed by 5-min USV recordings in a separate chamber. Four groups were defined across days: M-M, M-E, E-M, or E-E (M or E represent milk or ethanol infusion received each day). Lastly, all animals completed a 15-min ethanol intake test at PD 8 and immediately, pups were humanely sacrificed while brain samples were collected, homogenized, and frozen at −80°C to determine catalase activity. In all procedures, temperature was kept at 31–32°C through heating pads placed beneath the chambers. The overall factorial design of the present study was as follow: prenatal sc. administration (saline or D-penicillamine) × prenatal ig. administration (water or ethanol) × postnatal treatment (M-M, M-E, E-M, or E-E). Each of the 16 groups defined by this design was composed of no fewer than 16 pups with an equivalent sex representation. Figure 1 depicts the overall design of the present study.

### Artificial lactation test

At PDs 4 and 6, pups were removed from their maternal cages, intraorally implanted with a cannula made from 5-cm of polyethylene-10 tubing (PE-10, Clay Adams, Parsippany, NJ, USA) that allowed liquid infusions, and then they remained paired-house for 2 h in individual Plexiglas cages (25 cm × 25 cm) partially filled with clean corncob until evaluations took place. Shortly before each session, pups were anogenitally stimulated gently with a cotton swab to promote urination and defecation, weighed to the nearest 0.01 g/kg and individually placed in a plethysmograph chamber (see description below) where they received infusions of milk as a sole liquid (Milkaut skim milk, 1.5% fat) or milk mixed with 6% v/v ethanol (Porta Hnos, Cordoba, Argentina) for 40 min. Neonates received 8 intraoral infusions of the corresponding substance using an infusion pump (KD Scientific, Model 200, Holliston, MA, USA). Each liquid pulse was set to last 1 min while the inter-pulse duration was equivalent to 4 min, as previously described (Miranda-Morales et al., 2016; Acevedo et al., 2017a; D’aloisio et al., 2020). The overall amount of liquid infused was equivalent to 5.5% of the average pre-infusion body weight. Interpulse intervals and fluid volumes resemble the natural milk letdown in a non-anesthetized dam (Lincoln, 1973; Cramer and Blass, 1983; Hunt et al., 1993). Pre- and post-infusion weights were measured, percent body weight gain (% BWG) was calculated along with the absolute grams of ethanol per kilogram of body weight (g/kg) as an index of ethanol consumption early in the ontogeny. Similar procedures have been extensively used in prior studies (López and Molina, 1999; Arias and Chotro, 2006; Pautassi et al., 2008; Pueta et al., 2011; Acevedo et al., 2017a). In several studies are indicated that intraoral cheek canulation method allowed pups to control ingestion of fluids, including oral ethanol delivered (Arias and Chotro, 2006; Pautassi et al., 2008; Pueta et al., 2011; Miranda-Morales et al., 2016; Gaztañaga et al., 2017). The pups are able to control liquid intake by regulating taste reactivity responses (Arias and Chotro, 2005). The emission of mouthing and tongue protrusions helps ingest the fluid, whereas gaping, chin rubbing, and passive drips allow for partial avoidance of the solution (Arias et al., 2010).

### Determination of breathing frequencies and apneic episodes and latency to emit the first apneic response

Breathing frequencies and apneic episodes were recorded using a whole-body plethysmograph (Model 10G equipped with the software “Breath Medidor de Respiración,” Itcom, Argentina). It consists of two identical transparent and hermetic Plexiglas chambers (5 × 10 × 5 cm) interconnected via a polyurethane hose system that allows injection and extraction of equivalent amounts of air into the chambers keeping constant and equivalent pressures. One of the chambers is used as a testing device while the other servers as a reference box in terms of flow/air pressure rate. The plethysmograph records the rate differences between both chambers. These differences activate pressure sensor (AWM2100 Honeywell) that record one complete breathing episode every 1 × 10^–7^s. The apparatus registers the breathing frequencies every 1.5 s and the apneic episodes that were counted every time the airflow was interrupted for at least two normal respiratory cycles (0.5 s or more; Julien et al., 2010). Breathing frequencies were interpreted as mean breaths per minute and the total number of apneic episodes as well as latency to exhibit the first apneic episode were also assessed. In addition, a coefficient of breath/apneas was calculated at each bin of evaluation by using the number of breathing frequencies as the numerator per apneic episode as the denominator. For each session, unrestrained awake pups were introduced gently in the plethysmograph chambers as lids were closed. One basal minute was considerate to allow air pressure stabilization. These experimental procedures have been previously used to assess breathing patterns as function of ethanol exposure during pre- and postnatal period (Cullere et al., 2015; Macchione et al., 2016, 2018, 2021; Acevedo et al., 2017a,b; D’aloisio et al., 2020; Anunziata et al., 2021).

### Ultrasonic vocalizations test

Following each respiratory session at PDs 4 and 6, USVs were recorded throughout 5 min using a non-invasive ultrasound recording system ANL-817-1B (Med Associates, Inc. Georgia Regional Industrial Park, Swainsboro, GA, USA) analogous to the one used in Macchione et al. (2016). During the recording pups remained in separate Plexiglas cages (25 cm × 25 cm) inside soundproof boxes. The range of vocalizations attained was between 22 and 100 KHz as an indicator of emotional reactivity (Hofer, 1996; Molina et al., 2000). The total number of USVs within this range (22–100 KHz) were recorded.

### Ethanol intake test at PD 8

Similar procedures were followed as described in section “Artificial lactation test.” Before testing, pups were placed individually for 15 min in an open-field (20 cm in diameter and 16 cm in height) and received a continuous flow of a 6% (v/v) ethanol solution at a rate of 0.1 ml/min corresponding to 5.5% of their pre-infusion weight (Arias and Chotro, 2006). Animals could either consume or reject the infused fluid during the test. Post-infusion weights were registered, and percent body weight gain and absolute grams of ethanol per kilogram of body weight (g/kg) were calculated.

### Brain catalase activity

To measure brain catalase activity the entire brain was removed excluding the cerebellum and homogenized mixing phosphate buffer (50 mM; pH 7.0; 5 ml for each brain sample) with the protease inhibitor phenylmethylsulphonyl fluoride (PMSF; 0.01%; 50 μl for each brain sample). Immediately after, brain samples were spin-dried at 9,000 *g* for 10 min. Aliquots of supernatant were used to determine catalase activity levels (Aebi, 1984) and protein levels (Bradford, 1976) which varied for each animal. Catalase can degrade hydrogen peroxide (H_2_O_2_) which was measured directly by the decreased in absorbance during 5 min at 240 nm (ε_240_ = 0.0394 mmol-^1^ cm-^1^) via spectrophotometry (Aebi, 1984). The reaction kinetics of catalase activity was performed at room temperature (20°) using 909 μl phosphate buffer (50 mM; pH 7.0) containing 73 μl of H_2_O_2_ (150 mM) as a substrate and 18 μl brain supernatant (final volume of 1 ml). The resultant activity was expressed as units of catalase activity (mmol H_2_O_2_/min/mg of protein). Similar protocols to estimate catalase activity has been performed in previous studies (Correa et al., 2001; Klongpityapong et al., 2013; Mattalloni et al., 2013; Carvajal-Serna et al., 2020; Trujillo et al., 2020).

### Data analyses

Normal distribution assumptions were verified by Shapiro–Wilk’s and Leneve’s tests. The homogeneity of variances across independent groups defined by prenatal sc. administration (D-penicillamine or saline), prenatal ig. administration (ethanol or water) and postnatal treatment (milk or ethanol infusion) was assessed through Levene test. When considering apneic episodes and USVs at PDs 4 and 6 as well as brain catalase activity at PD 8 based on prenatal treatment (sc. and ig.) and postnatal treatment, homogeneity of variance was not found. Therefore, the data from apneic episodes and USVs was transformed using a natural logarithmic transformation [bij = log (apneas or ultrasonic vocalizations + 1)]. This strategy achieves the criterion of homogeneity of variance across independent groups (McCune et al., 2002; Feng et al., 2014) as is also achieved after square root transformation for brain catalase activity. This last transformation is useful when the data is likely to be Poisson distributed rather than normally distributed (Sokal and Rohlf, 1994). Preliminary analyses indicated that sex did not exert significant main or interaction effects in any analyses of this study, therefore all inferential analyses were performed by collapsing sex across treatments.

Breathing frequencies, apneic episodes, coefficient breaths/apneas and USVs were analyzed using five-way mixed analysis of variance (ANOVA). Prenatal sc. administration, prenatal ig. administration and postnatal treatment (see methods section, M-M; M-E; E-M or E-E) served as between factors while days of assessment (PDs 4 or 6) and bins or minutes of evaluation (bins 1–15 for breathing frequencies and apneic episodes, minutes 1–5 for USVs) were considered as within-group variables. A four-way mixed ANOVA (prenatal sc. administration × prenatal ig. administration × postnatal treatment × days of assessment) was employed to analyze latency to emit the 1st apneic episode. To better understand the apneic episodes and USVs as function of postnatal treatment, follow-up repeated measures ANOVAs were conducted based on the values assessed on the second day of assessment (PD 6). For more details, please see “Results” section.

Percentage body weight gain (% BWG) scores during PDs 4 and 6 were analyzed via a four-way mixed ANOVA, where prenatal sc. administration, prenatal ig. administration and postnatal treatment served as independent factors and days of assessments as repeated measures. A three-way mixed ANOVA was performed to analyze ethanol intake as absolute grams of ethanol per kilogram (g/kg) during PDs 4 and 6. The between factors were prenatal sc. administration and prenatal ig. administration while days of assessments served as the within-measure factor. In addition, a separate three-way ANOVA (prenatal sc. administration × prenatal ig. administration × times exposed to ethanol –1 or 2-) was conducted to analyze absolute grams of ethanol at PD 6.

Percentage body weight gain, ethanol intake (g/kg), and brain catalase activity at PD 8 were analyzed via a three-way ANOVA. Prenatal sc. administration, prenatal ig. administration and postnatal treatment served as the between factors. Additionally, Pearson’s correlation coefficients were calculated to determine the strength of the association between ethanol intake (% BWG or g/kg of ethanol) and brain catalase activity at PD 8.

Data corresponding to pup body weights across days was analyze via a repeated measures ANOVA. It was performed considering days of assessment as a function of prenatal ig. administration.

Following the execution of the ANOVAs, each significant main effect or significant interactions were further examined using Duncan *Post hoc* test (*p* < 0.05). The statistical analyses were performed using the STATISTICA 8.0 software. Results are reported as the mean ± SEM.

## Results

### Pup body weights

Pup body weights indicated a significant main effect of days of assessment *F*(2,514) = 16424, *p* < 0.001. As expected, body weights significantly increased as a function of age. Consistent with previous studies (Abate et al., 2014; Cullere et al., 2015), prenatal ig. administration of ethanol did not exert teratological effects *F*(1,255) = 1.16, *p* = 0.28.

### Breathing frequencies

**FIGURE 2:**
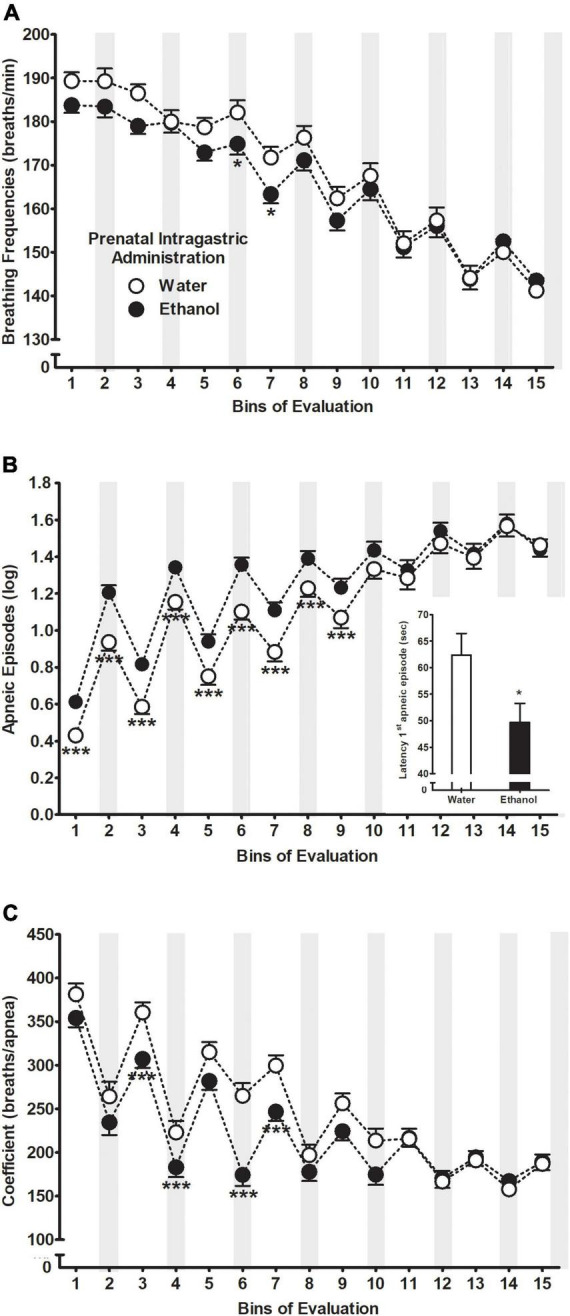
**(A)** Breathing frequencies, **(B)** apneic episodes, and **(C)** total number of breaths until an apnea occurrence (coefficient: breaths/apneas) as a function of prenatal intragastric administration of ethanol (black) or water (white) across bins of evaluation. Gray bars indicate intraoral infusions during the artificial lactation test. *p < 0.05 and ***p < 0.001 significant differences between groups within a time point. Data were collapsed across postnatal days of assessment. The latter did not exert a significant main effect or significantly interact with the remaining variables. Apneic episode values were transformed using a natural logarithmic transformation [bij = log (apneic episode + 1)]; please see “Data analyses” section. Vertical lines illustrate standard errors of the mean (SEMs).

Figure 2A illustrates the average breathing frequencies across bins of evaluation as a function of prenatal ig. administration. The ANOVA indicated a significant main effect of bins of evaluation *F*(14,3416) = 132.25, *p* < 0.001 as well as a significant interaction between prenatal ig. administration and bins of evaluation *F*(14,3416) = 1.83, *p* = 0.03. According to Duncan’s *post hoc* tests and regardless of prenatal treatment, breathing frequencies spiked from bin 6 to 15 every time the liquid pulse (i.e., 1-min) took place compared to those bins when no liquid infusion was delivered (i.e., 4-min interpulse). Moreover, a transient decrease of breathing frequencies was observed from bin 6 to the end of the evaluation. At bins 6 and 7, those pups exposed to ethanol during the gestational period showed a significant decrease in terms of respiration frequencies relative to those treated with water.

### Apneic episodes

The between-within ANOVA (prenatal sc. administration × prenatal ig. administration × postnatal treatment × days of assessment × bins of evaluation) for apneic episodes indicated significant main effects of prenatal ig. administration *F*(1,244) = 7.70, *p* = 0.006, bins of evaluation *F*(14,3416) = 175.44, *p* < 0.001 as well as an interaction between both factors *F*(14,3416) = 4.29, *p* < 0.001 depicted in Figure 2B. Duncan’s comparisons indicated that those pups exposed to ethanol during gestation showed more apneas compared to those pups exposed to water almost at all given bins of evaluation (1–9). Similar to breathing frequencies, apneic episodes were higher during those bins where the intraoral infusions occurred compared to the remaining bins. Moreover, those animals prenatally exposed to ethanol showed a shorter latency to emit the first apneic episode compared to those pups exposed to water, *F*(1,244) = 5.52, *p* = 0.02.

Similarly, the ANOVA of the coefficient breaths/apneas showed significant main effects of prenatal ig. administration *F*(1,244) = 8.08, *p* = 0.005 and bins of evaluation *F*(14,3416) = 74.75, *p* < 0.001 following by the interaction between both factors *F*(14,3416) = 3.89, *p* < 0.001. Briefly stated, Duncan tests indicated a transient decrease of the coefficient throughout the evaluation with smaller scores when animals received intraoral infusions compared to the 4-min interpulse intervals. In addition, the number of breathing frequencies until an apneic episode occurred was significantly lower at bins 3, 4, 6, 7, and 10 in the group of animals that received ethanol during gestation relative to those that received water. Please see Figure 2C.

**FIGURE 3:**
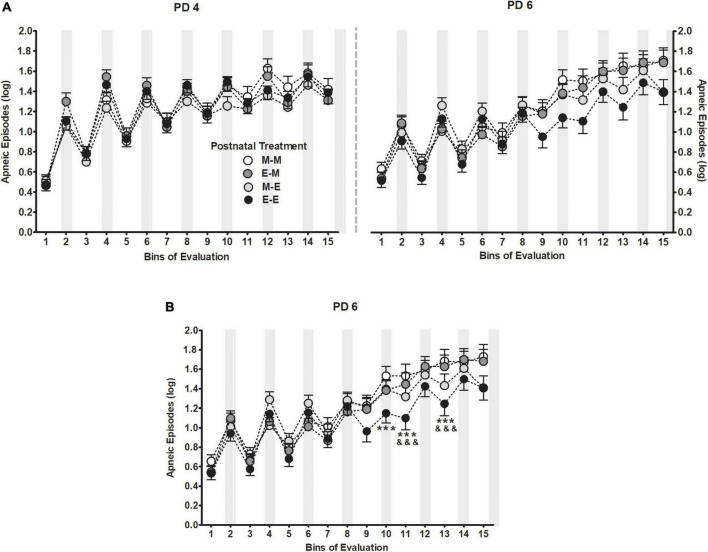
Apneic episodes (log) [bij = log (apneic episode + 1)] as a function of postnatal treatment across bins of evaluation **(A)** during PDs 4 and 6 **(B)** and at PD6. Gray bars indicate intraoral infusions during the artificial lactation test. Black dots represent pups that received ethanol as the sole liquid (E-E) both days, dark gray dots represent pups exposed to milk mixed with ethanol at PD4 and switched to milk as the sole liquid at PD6 (E-M), light gray dots represent pups initially exposed to milk and switched to milk mixed with ethanol at PD6 (M-E), white dots represent pups that received milk as the sole liquid (M-M) both days. Differences at PD6 between E-E and M-M groups (***p < 0.001), and between E-E and E-M groups (^&&&^*p* < 0.001) within a time point. Vertical lines illustrate standard errors of the mean (SEMs).

Apneic episodes during PDs 4 and 6 were also affected as a function of postnatal treatment. The five-way between-within ANOVA indicated a significant interaction among postnatal treatment, days of assessment and bins of evaluation *F*(42,3416) = 1.46, *p* = 0.03 as it is shown in Figure 3A. To better understand the triple interaction, a separate repeated measures ANOVA was performed considering apneic episodes as function of postnatal treatment across bins of evaluation at PD 6. The ANOVA yielded a significant interaction between both factors *F*(42,3584) = 2.06, *p* < 0.001 indicating that pups postnatally exposed twice to milk mixed with ethanol (E-E) showed significantly fewer apneas (i.e., bins 10–11, and 13) than those pups that received milk as the sole liquid (M-M), and relative to those pups in the E-M group at bins 11 and 13. No differences were observed between E-E and M-E animals. Please see Figure 3B.

### Artificial lactation test

**FIGURE 4:**
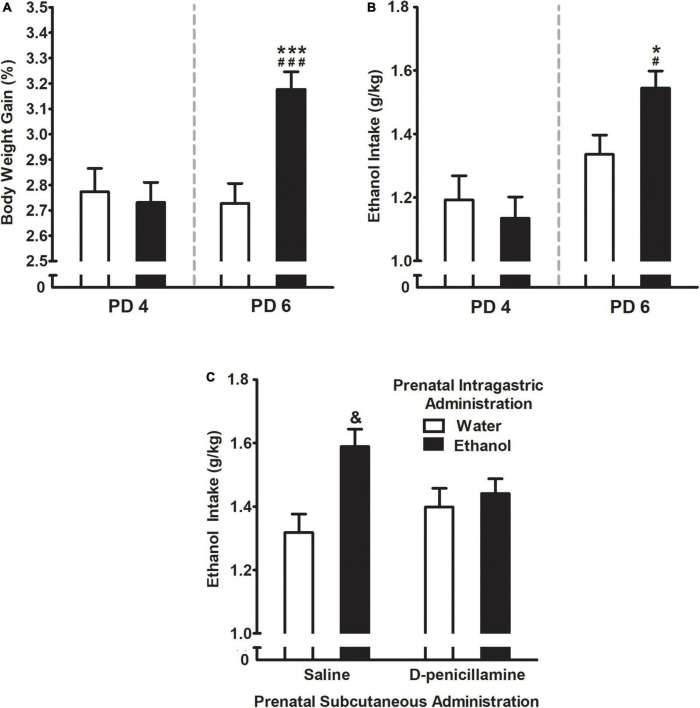
**(A)** Percent body weight gain and **(B)** ethanol intake (g/kg) as a function of prenatal intragastric administration of ethanol (black) or water (white), and postnatal days of assessment (PDs 4 and 6). ^###^*p* < 0.001, ^#^*p* < 0.05 indicates that values at PD6 are significantly different from PD 4 in the group of pups prenatally exposed to ethanol. ***p < 0.001, *p < 0.05 indicate the difference between the group exposed to ethanol relative to water group at PD6. **(C)** Ethanol intake (g/kg) as a function of prenatal intragastric and subcutaneous administration. ^&^*p* < 0.05 indicates pups prenatally exposed to ethanol are different from all groups. Vertical lines illustrate standard errors of the mean (SEMs).

Intake test assessed by percent body weight gain (% BWG) was significantly affected by prenatal ig. administration *F*(1,244) = 5.43, *p* = 0.02 and days of assessment *F*(1,244) = 7.62, *p* = 0.006. Similarly, ethanol intake as the absolute grams of ethanol per kilogram of body weight (g/kg) was affected by the days of assessment *F*(1,61) = 22.72, *p* < 0.001. Both, % BWG and absolute grams of ethanol (g/kg) showed an interaction between these factors [*F*(1,244) = 11.49, *p* = 0.0008 and *F*(1,61) = 5.28, *p* = 0.03; respectively]. More in detail, Duncan’s *post hoc* test indicated that regardless of the properties of the liquid intraorally infused, all animals drank roughly the same amount of fluid during the first day of assessment (i.e., PD 4), but those prenatally exposed to ethanol increased their % BWG as well as ethanol intake (Figures 4A,B; respectively) during the second day of assessment (PD 6). The water-exposed controls, by contrast, remained on the same drinking pattern as the previous day of assessment.

Three-way ANOVA (prenatal sc. administration × prenatal ig. administration x times exposed to ethanol –1 or 2-) yielded a significant interaction comprising prenatal sc. administration and prenatal ig. administration *F*(1,211) = 4.38, *p* = 0.04. As shown in Figure 4C, pups prenatally exposed to ethanol consumed significantly more ethanol (g/kg) relative to their control groups. However, this effect was attenuated by the administration of D-penicillamine during late gestation. In addition, pups prenatally exposed to ethanol and administrated with D-penicillamine were similar to control groups in terms of ethanol consumption.

### Ultrasonic vocalizations test

In terms of USVs, the corresponding five-way between-within ANOVA (prenatal sc. administration × prenatal ig. administration × postnatal treatment × days of assessment × minutes of evaluation) indicated significant main effects of postnatal treatment *F*(3,242) = 5.56, *p* = 0.001 and minutes of evaluation *F*(4,968) = 10.03, *p* < 0.001. The following significant interactions were also observed: prenatal ig. administration × days of assessment × minutes of evaluation *F*(4,968) = 2.94, *p* = 0.020, and postnatal treatment × minutes of assessment *F*(3,242) = 10.07, *p* < 0.001.

**FIGURE 5:**
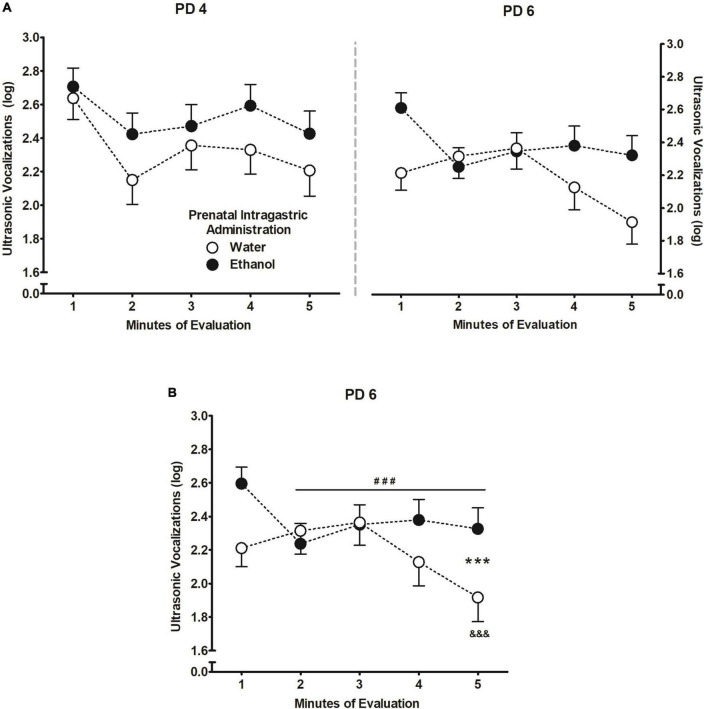
Ultrasonic vocalizations (log) [bij = log (ultrasonic vocalizations + 1)]. **(A)** During PDs 4 and 6. **(B)** As a function of prenatal intragastric administration of ethanol (black) or water (white) across minutes of evaluation at PD6. ^###^*p* < 0.001 indicates that pups prenatally exposed to ethanol exhibited significantly fewer vocalizations from the second minute of evaluation relative to the first minute and remained steady from minutes 3–5. ^&&&^*p* < 0.001 refers to prenatally water-treated group exhibited fewer vocalizations during the last-minute relative to the first 4 min of evaluation. ***p < 0.001 represents the differences between pups prenatally exposed to ethanol and those water-treated within a time point. Vertical lines illustrate standard errors of the mean (SEMs).

Figure 5A depicts the triple interaction among factors, however, to better understand the results, a follow-up repeated measures ANOVA was performed based on prenatal ig. administration and minutes of evaluation at PD 6. The ANOVA revealed a main effect of minutes of evaluation *F*(4,1024) = 4.27, *p* = 0.002, and the interaction between prenatal ig. administration and minutes of evaluation *F*(4,1024) = 4.59, *p* < 0.001. Pups prenatally exposed to ethanol exhibited significantly fewer USVs from the second minute and remained steady throughout the evaluation, relative to the first minute. Except for the last minute of evaluation when USVs significantly decreased, pups prenatally exposed to water elicited similar USVs to ethanol-exposed pups during the first four minutes of evaluation (Figure 5B).

**FIGURE 6:**
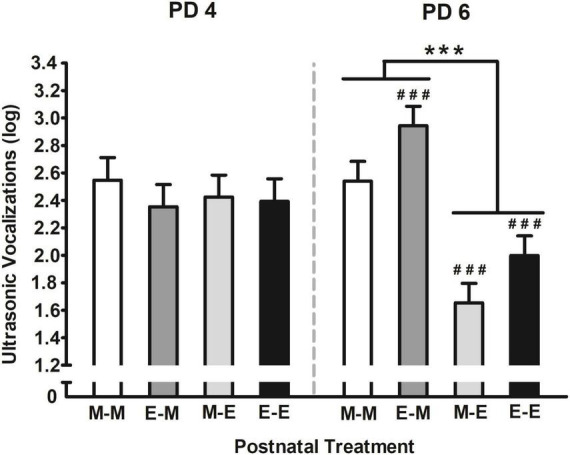
Ultrasonic vocalizations (log) [bij = log (ultrasonic vocalizations + 1)] as a function of postnatal treatment and days of assessment. Pups were exposed to intraoral infusions of either milk (M), or ethanol mixed with milk (E) on each day of assessment (PDs 4 or 6). Four groups were defined as a function of these infusions across days: M-M, M-E, E-M, or E-E. ^###^*p* < 0.001 indicates that vocalizations on PD 6 are significantly different from those observed on PD 4 at each given group. ***p < 0.001 indicates the differences among the groups that received ethanol mixed with milk (E-E and M-E groups) relative to those that received milk (E-M and M-M) at PD6. Vertical lines illustrate standard errors of mean (SEMs).

The interaction comprising postnatal treatment and days of assessment is depicted in Figure 6. According to Duncan’s *Post hoc* tests, USVs were similar among groups during the first day of assessment (PD 4), however, those pups that postnatally received ethanol mixed with milk at PD 6 (i.e., M-E and E-E) significantly emitted fewer vocalizations. In contrast, those pups that received milk and had prior experienced ethanol mixed with milk (E-M), emitted more USVs the second day than the previous day of assessment. Furthermore, those pups intraorally infused with ethanol at PD 6 (i.e., M-E and E-E) had significantly fewer USVs than those pups exposed to milk either one or two times (i.e., M-M and E-M).

### Ethanol intake test

**FIGURE 7:**
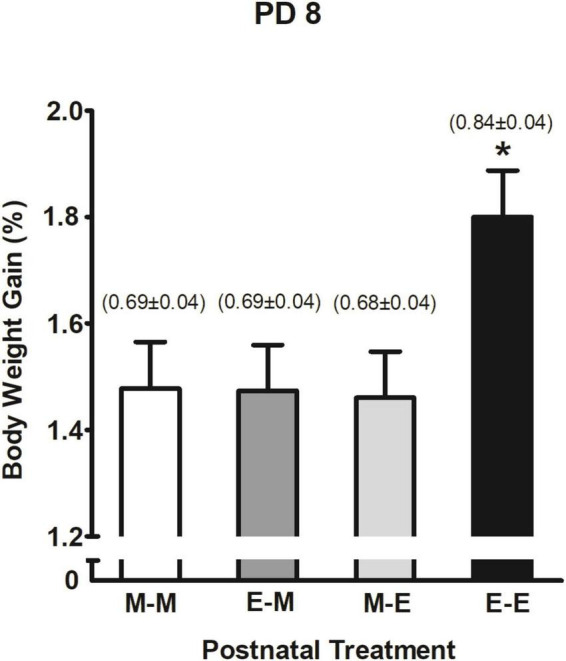
Percent body weight gain as a function of postnatal treatment at PD 8. *p < 0.05 indicates pups that received ethanol as the sole liquid (E-E) are different from all groups. Numbers in brackets indicate ethanol intake (g/kg) at PD 8 (mean ± SEM). Vertical lines illustrate standard errors of the mean (SEMs).

In terms of % BWG and ethanol intake (g/kg) the corresponding three-way ANOVA (prenatal sc. administration × prenatal ig. administration × postnatal treatment) showed a significant main effect of postnatal treatment *F*(3,244) = 3.56, *p* = 0.015. As shown in Figure 7, pups that received ethanol mixed with milk twice (E-E) significantly increased their % BWG and ethanol intake relative to groups that postnatally received milk at least once (M-M, M-E, and E-M). The latter three groups expressed similar outcomes.

### Brain catalase activity

**FIGURE 8:**
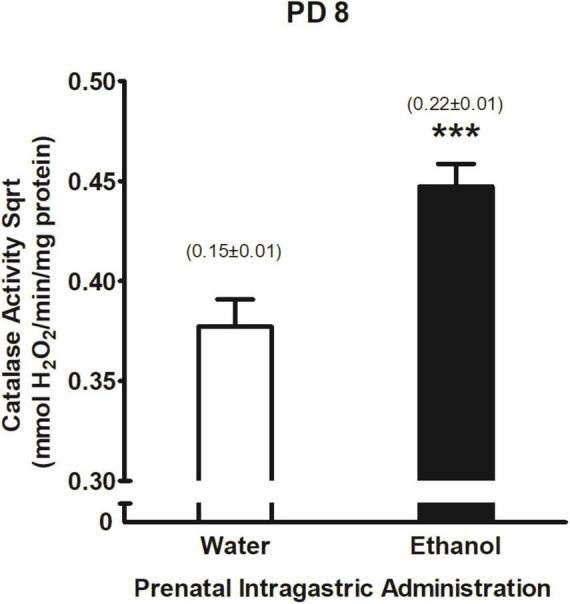
Catalase activity Sqrt (mmol H_2_O_2_/min/mg protein) as a function of prenatal intragastric at PD 8. ***p < 0.001 indicates that pups prenatally exposed to ethanol are different from those in the water-treated group. Catalase activity values were transformed using a square root transformation. Numbers in brackets indicate catalase values without transformation (mean ± SEM). Vertical lines illustrate standard errors of the mean (SEMs).

Activity of central catalase system was found to significantly vary as a function of prenatal ig. administration *F*(1,214) = 15.75, <0.001. Duncan’s *Post hoc* tests showed that pups exposed to ethanol during late gestation had higher levels of catalase activity relative to pups that were exposed to water (please see Figure 8). According to Pearson correlation coefficient, neither ethanol intake nor catalase brain activity were associated (*r* = 0.07, *p* > 0.05) nor neither each group defined by the overall design of the study (all *p*’s > 0.01).

## Discussion

The present study examined the impact of brief prenatal and postnatal experience with ethanol upon respiratory consequences, affective state, and ethanol intake during the first week of the rat. Ethanol’s first metabolite role was assayed along with the activity of brain catalase system during this stage of development. In line with previous findings (Cullere et al., 2015; Acevedo et al., 2017a; Macchione et al., 2018, 2021), we observed that early exposure to ethanol sensitizes pups to ethanol’s respiratory depressant effects upon breathing patterns and increases apneic episodes (Figures 2A,B). In addition, pups showed lower breathing frequencies until an apneic episode occurred (coefficient breaths/apneas, Figure 2C). Regardless of the treatment’s nature, a transient decrease in breathing frequencies (Figure 2A) was observed throughout evaluations, indicative of a habituation process (Macchione et al., 2016, 2018, 2021; Acevedo et al., 2017a,b; D’aloisio et al., 2020; Trujillo et al., 2020). The short and long-term effects of ethanol on neonatal respiratory plasticity more recently have received attention given the association between fetal alcohol exposure and the sudden infant death syndrome (Iyasu et al., 2002; Burd et al., 2004; Bailey and Sokol, 2011; O’Leary et al., 2013). Preclinical studies have indicated that chronic ethanol exposure throughout gestation and lactation period, are responsible for depressing breathing frequencies, disrupting the central respiratory network and suppressing the adaptive response to low oxygen environment in neonates, infants, and adults (Dubois et al., 2006, 2008, 2013; Kervern et al., 2009; Dubois and Pierrefiche, 2020). However, while effects on the respiratory system seems due to chronic exposure to ethanol, our data along with recent studies show that brief fetal and neonatal experiences with a moderate ethanol dose are sufficient to functionally disrupt the respiratory plasticity of the organisms under associative and non-associative learning processes (Cullere et al., 2015; Macchione et al., 2016, 2018, 2021; Acevedo et al., 2017a; D’aloisio et al., 2020; Anunziata et al., 2021).

Relative to ethanol-induced apneic episodes and given the relevance of stress-like events that may impact respiratory plasticity (Macchione et al., 2018) the question arises as to whether these physiological disruptions may reflect, at least in part, an anxiety state of the organisms. For instance, chronic prenatal ethanol exposure alters the HPA axis tone by inducing a long-term hyperresponsiveness toward stressors during adulthood (Weinberg et al., 2008; Lan et al., 2009). Animals express an overall increase in anxiety-like behaviors such as reduced time spent in the open arms of an elevated plus-maze, in the center of a hole-board arena, as well as an extended latency to eat in a novelty-suppressed feeding test (Hofmann et al., 2005; Hellemans et al., 2010; Cullen et al., 2013). Yet, the former studies involve weaning or older animals whilst relatively little is known about ethanol anxiogenic proprieties during early stages of life. In our study, brief experiences with a moderate ethanol dose during the last 4 days of gestation increased apneic episodes and shortened the latency to emit the first apneic episode relative to controls (Figure 2B). In addition, those pups that received mixed milk with ethanol twice (i.e., E-E) showed significantly fewer apneic episodes compared to the groups that received milk as the sole liquid (i.e., M-M and E-M). The latter findings go in line with previous studies indicating that the mere -central or peripheral- experience with ethanol during PDs 3–9 attenuates apneic episodes induced by stress-related factors, suggesting early ethanol’s anxiolytic effects if the experience with the drug occurs postnatally (D’aloisio et al., 2020; Trujillo et al., 2020; Anunziata et al., 2021). Given the scarce pup’s behavioral repertoire and the lack of tests adjusted to age, both apneic episodes and latency to emit them may serve as proxies for animals’ anxiety levels.

Alike apneic episodes, USVs is a sensitive and useful tool when assessing innate anxiety in socially isolated pups. Moreover, pharmacological approaches have shown to affect USV emission response in different directions, while anxiolytic drugs decrease vocalization rate, anxiogenic drugs seem to increase it (Hofer, 1996; Branchi et al., 2001; Dirks et al., 2002; Hofer et al., 2002). Similar outcomes are observed when analyzing USV emission following ethanol exposure. For instance, chronic ethanol exposure to 30% throughout gestation increases USV emission in infant and juvenile rats (Shahrier and Wada, 2018, 2020), whereas postnatally the use of a “binge” model of ethanol exposure to 3.0 or 6.0 g/kg (Barron et al., 2000; Barron and Gilbertson, 2005) as well as a low 0.5 g/kg ethanol doses are effective to reduce vocalizations when pups are evaluated sober (Barron et al., 2000; Barron and Gilbertson, 2005) or 5 min after receiving the drug (Pautassi et al., 2007). Similarly, when we assessed the total number of USVs from a wide frequencies range (22–100 kHz), pups increased their vocalizations if they had experienced a moderate ethanol dose (2.0 g/kg) during the last 4 days of gestation compared to animals that had been exposed to water during the same period (Figure 5B). Conversely, pups exposed to ethanol postnatally decreased USVs while are intoxicated relative to sober pups (Figure 6). Results suggest that a brief moderate ethanol exposure may exert both anxiogenic or anxiolytic effects in terms of USVs and the latter seem closely related to the nature and the period of development in which such exposure happens. As has also been indicated for other factors such as dose and post-administration time which appear to exert differential properties of ethanol during early ontogeny (Pautassi et al., 2006, 2007). Moreover, USVs shared a similar pattern of response to what we observed in apneic episodes, whereas changes depended on the moment ethanol experience took place (Figures 2B, 3). However, we observed no correlation between the apneic episodes and USVs. It is likely that since the elapsed time between each assessment was 40-min apart, may had hampered our chance to trace and understand the effects of ethanol intraoral infusions; hence, limiting our capacity to estimate the relative contribution of both variables.

Under the present experimental conditions, we were unable to observe a role of gestational acetaldehyde upon respiratory plasticity or affective state of the organisms. To our knowledge, there is one study that reports respiratory depressions following acetaldehyde central administration 5 minutes, but not 60 or 120 after (Acevedo et al., 2017b). As to the role of acetaldehyde in the modulation of ethanol’s affective state, the results in adult rodents are controversial and seem to depend on factors such as the dose, route of administration, or animal strain. For instance, rats’ acetaldehyde intake in a two-bottle choice test (Plescia et al., 2015) and rats or mice administration of the metabolite (100 mg/kg, ip) reduce the time spent in the open arms of an elevated plus-maze and in the center of an open field (Correa et al., 2005; Escrig et al., 2012), indicating an acetaldehyde-induced anxiogenic effect. In contrast, over a wide dose range (30–170 mg/kg) peripheral acetaldehyde as its pharmacological inhibition (cyanamide) failed to exert anxiolytic or anxiogenic effects in C57BL/6J mice assessed in the elevated plus-maze (Quertemont et al., 2004; Tambour et al., 2005). However, central acetaldehyde infusion in rats (e.g., 61.7 μg) induced anxiolytic consequences in the same test (Correa et al., 2005), and its inactivation (D-penicillamine; 50 mg/kg) blocked the anxiolytic properties of ethanol not only in the elevated plus maze but also in the light/dark box (Correa et al., 2008). The latter study acetaldehyde was inactivated 30 min before each test took place, on the contrary, in our study we exposed animals to D-penicillamine during the late gestation and postnatally evaluated its effects. It is likely that the time span between these two periods prevented us from appropriately examining either respiratory or affective consequences of the metabolite. Nevertheless, a 50 mg/kg D-penicillamine dose that has shown efficacy in reducing central levels of acetaldehyde from ethanol metabolism (Serrano et al., 2007) is not effective in preventing the anxiogenic effects generated by a high dose of acetaldehyde in adult mice (100 mg/kg, ip) (Escrig et al., 2007). Unfortunately, we were unable to measure the levels of acetaldehyde that circulated in the dam’s amniotic fluid, or the amount of metabolite effectively sequestered by D-penicillamine. If D-penicillamine did not fail to inactivate the acetaldehyde *in utero* we can hypothesize that the metabolite does not contribute to the effect of ethanol upon respiratory response or the affective state of the organisms, at least not, under the present experimental protocol.

Gestational acetaldehyde did appear to mediate subsequent ethanol intake on the second day of assessment when using an artificial lactation test (Figure 4C). Those pups prenatally exposed to a moderate ethanol dose showed a higher %BWG and absolute ethanol intake than controls (Figures 4A,B) as also reported in the short- and long-term (Domínguez et al., 1998; Pueta et al., 2008; Díaz-Cenzano and Chotro, 2010; Fabio et al., 2013, 2015); an event recently proposed as fetal early programming of subsequent ethanol affinity (Miranda-Morales et al., 2020). Along with a heightened ethanol intake, pups showed an overall increase in milk consumption -a natural reinforcer- (Bachmanov et al., 2003; Pueta et al., 2008). Both rat and human infants are capable of detecting minimal amounts of ethanol in the milk (Mennella and Beauchamp, 1991; Mennella, 1997; Pepino et al., 1998). In humans, it is likely that alcohol remains present in those breastfeeding women who consumed alcohol while pregnant, as such continue to expose their children to the effects of ethanol after giving birth (Jacobson, 1997; Kim, 2019). Furthermore, when acetaldehyde was sequestered via D-penicillamine ethanol intake decreased at similar levels to controls (Figure 4C). The role of acetaldehyde in ethanol consumption had been explored mainly in adult rodents, where the central acetaldehyde administration increased self-administration (Amit et al., 1977) and reduced operant behaviors when given peripherally (McLaughlin et al., 2008). Moreover, the absence of acetaldehyde via D-penicillamine decreases ethanol consumption, operant self-administration (Font et al., 2006; Peana et al., 2015), and prevents alcohol relapse-like drinking as well (Orrico et al., 2013). It has been proposed that peripheric or central production of acetaldehyde could modulate the sensitivity toward ethanol aversive or appetitive motivational proprieties, respectively (Peana et al., 2016). Despite the lack of more studies that aim to understand ACD’s contribution to ethanol’s reinforcing effects early in development, it has been reported that the dose of D-penicillamine we used (50 mg/kg), is capable of blocking appetitive conditioning in neonates and conditioned place preference in infant rats (Pautassi et al., 2011; March et al., 2013b; respectively). In line with Gaztañaga et al. (2017) study assessed during infancy, our findings showed that D-penicillamine administration during the last 4 days of gestation decreases pups’ ethanol intake in terms of %BWG during the first week after birth. This acquires relevance when considering the particular characteristics of early ontogeny in regard to central and peripheral ethanol metabolization to its first metabolite, acetaldehyde (Del Maestro and McDonald, 1987; Kelly et al., 1987). During early ontogeny, a peak in brain catalase system activity is expected, and low or lack of peripheral metabolization rates given the hepatic immaturity of the organisms, helping to better understand the scenario of ethanol’s appetitive effects mainly during this developmental period (March et al., 2013a).

Indeed, we observed that brain catalase system activity was increased as a function of gestational ethanol exposure (Figure 8). About 60% of this enzymatic system is mainly responsible for metabolizing ethanol into acetaldehyde in the brain (Aragon et al., 1991b; Zimatkin et al., 2006) and its peak levels of activity occurs from late gestational period to the first week after birth (Del Maestro and McDonald, 1987). It seems that repeated experiences with high ethanol doses are necessary to induce such brain catalase activity (Hamby-Mason et al., 1997; Trujillo et al., 2020). Not only high but also moderate ethanol exposures, as we observed here, activate the enzymatic system. While brain catalase activity and voluntary ethanol intake correlate in adult rats (Amit and Aragon, 1988) in pups, we failed, as in Trujillo et al. (2020), to observe such correlation. Unfortunately, we lacked a direct measure of ethanol levels prior catalase determination at PD 8. It is worth noting as in Holbrook et al. (2022), the correlation between catalase activity and ethanol intake may sometimes be positive (Aragon et al., 1985; Karahanian et al., 2011), negative (Gill et al., 1996; He et al., 1997) or null (He et al., 1997). Altogether, results seem to indicate that central catalase activity depends, at least in part, on repeated exposures to ethanol. On a molecular level, brain catalase system activity enhance has been considered as a biomarker of adaptation to stress mechanisms (Röhrdanz et al., 2001; Sen et al., 2003; den Besten et al., 2013; Gonchar et al., 2018) and it is believed to protect the embryo from teratological effects of fetal alcohol exposure (Abramov and Wells, 2011a,b; Abramov et al., 2012; Miller et al., 2013; Wells et al., 2016). However, more studies are needed to shed light on these and other possible arguments.

As a whole the present study indicates that brief moderate ethanol-related experience during the late gestation is enough to disrupt respiratory plasticity of the organisms, to increment subsequent ethanol intake and to induce brain catalase activity. Also, repeated postnatal experience with ethanol increments ethanol intake. Moreover, early experience with ethanol generates differential affective states in apneic episodes and USVs depending on the moment the exposure to ethanol occurs (prenatal or postnatal). Within the body of results, acetaldehyde contributes uniquely to the positive reinforcing effect of ethanol on subsequent drug intake.

## Data availability statement

The original contributions presented in this study are included in the article, further inquiries can be directed to the corresponding authors.

## Ethics statement

This animal study was reviewed and approved by the Institutional Animal Care and Use Committee (CICUAL-INIMEC-CONICET-UNC). All experimental treatments and animal’s maintenance were in compliance with the Guide for Care and Use of Laboratory Animals (National Research Council, 2011).

## Author contributions

JM and GD’a contributed to the conception and design of the work. GD’a, MA, and JM contributed to the analysis and interpretation of data. GD’a and MA participated in the writing and revision of the first draft, supervised by JM. GD’a and AA-A conducted the experimental procedures. VT provided the technical support and oversaw brain catalase determination. All authors critically reviewed the content and approved the final version for publication.
